# Lycopene Inhibits Toll-Like Receptor 4-Mediated Expression of Inflammatory Cytokines in House Dust Mite-Stimulated Respiratory Epithelial Cells

**DOI:** 10.3390/molecules26113127

**Published:** 2021-05-24

**Authors:** Jiyeon Choi, Joo Weon Lim, Hyeyoung Kim

**Affiliations:** Department of Food and Nutrition, College of Human Ecology, Yonsei University, Seoul 03722, Korea; chlwldus6204@hanmail.net (J.C.); jwlim11@yonsei.ac.kr (J.W.L.)

**Keywords:** cytokines, house dust mite, lycopene, Toll-like receptor 4, reactive oxygen species, respiratory epithelial cells

## Abstract

House dust mites (HDM) are critical factors in airway inflammation. They activate respiratory epithelial cells to produce reactive oxygen species (ROS) and activate Toll-like receptor 4 (TLR4). ROS induce the expression of inflammatory cytokines in respiratory epithelial cells. Lycopene is a potent antioxidant nutrient with anti-inflammatory activity. The present study aimed to investigate whether HDM induce intracellular and mitochondrial ROS production, TLR4 activation, and pro-inflammatory cytokine expression (IL-6 and IL-8) in respiratory epithelial A549 cells. Additionally, we examined whether lycopene inhibits HDM-induced alterations in A549 cells. The treatment of A549 cells with HDM activated TLR4, induced the expression of IL-6 and IL-8, and increased intracellular and mitochondrial ROS levels. TAK242, a TLR4 inhibitor, suppressed both HDM-induced ROS production and cytokine expression. Furthermore, lycopene inhibited the HDM-induced TLR4 activation and cytokine expression, along with reducing the intracellular and mitochondrial ROS levels in HDM-treated cells. These results collectively indicated that the HDM induced TLR4 activation and increased intracellular and mitochondrial ROS levels, thus resulting in the induction of cytokine expression in respiratory epithelial cells. The antioxidant lycopene could inhibit HDM-induced cytokine expression, possibly by suppressing TLR4 activation and reducing the intracellular and mitochondrial ROS levels in respiratory epithelial cells.

## 1. Introduction

House dust mites (HDM) are recognized as a critical cause of allergic disorders [1,2]. Among HDM, *Dermatophagoides farinae*, *Dermatophagoides pteronyssinus,* and *Euroglyphus maynei* are the most abundant mites responsible for the production of HDM allergens, which is a common cause of allergic rhinitis and asthma. The predominant species of HDM differ by location, and *D. farinae*, the American house dust mite, is the dominant species in Korea [3]. Recently, we showed that the extract of *D. farinae* increases the levels of reactive oxygen species (ROS), the activation of nuclear factor κB (NF-κB), and IL-8 expression in respiratory epithelial H292 cells [4]. 

The protease activities of HDM allergens target tight junction proteins such as occludin and claudins and thus, increase the barrier permeability of the airway mucosa [5] and subsequent influx of allergens [5,6]. HDM cleaved tight junction protein ocludin and thus, increased transepithelial influx after 2 h, which continued until 7 h-incubation [5]. These studies show that HDM directly enter into airway epithelium and affect cell organelles. In addition to increasing epithelial cell permeability, HDM proteases trigger and stimulate Toll-like receptor 4 (TLR4) in epithelial cells [7,8,9]. Moreover, HDM increase NADPH oxidase dual oxidase 1 (DUOX1) and increase ROS production in airway epithelium [10]. 

Reactive oxygen species (ROS) activate the signaling of TLR4 in non-small cell lung cancer (NSCLC) cells induced by lipopolysaccharide (LPS) stimulation [11]. ROS regulate TLR4-mediated activation of NF-κB κ and IL-8 expression in the monocyte-like cell line THP-1 [12]. TLR4 is a membrane-bound member of the TLR family of pattern-recognition receptor [13]. TLR4 induces an innate immune response through transcriptional regulation, leading to the production of pro-inflammatory cytokines [14,15,16]. Taken together, we can postulate that HDMs activate NADPH oxidase and produce ROS, which stimulates TLR4 signaling, leading to activation of NF-κB and cytokine expression

Interleukin-6 (IL-6) is a pro-inflammatory cytokine that plays a key role in acute-phase response and the transition from acute to chronic inflammation [17,18]. Several studies have shown that the dysregulation of IL-6 production is a major contributor to the pathogenesis of chronic inflammatory and autoimmune diseases [19,20]. Interleukin-8 (IL-8) is a chemokine that is induced by diverse inflammatory stimuli in various cells, including monocytes, macrophages, fibroblasts, and endothelial cells [21]. IL-8 is known as a key factor in localized inflammation, as its secretion can be increased by oxidative stress, thereby leading to the recruitment of inflammatory cells and a further increase in oxidative stress mediators [22]. The increased expression of selected cytokines, such as IL-6 and IL-8, can eventually promote inflammation in the body, including respiratory epithelium [23].

Lycopene is a lipid-soluble antioxidant and a member of the carotenoid family of phytochemicals. It can be synthesized by many plants and microorganisms, but not by animals or humans, and is found in some red-colored vegetables and fruits, such as tomatoes [24,25]. Lycopene has a highly unsaturated open straight-chain hydrocarbon structure consisting of eleven conjugated and two unconjugated double bonds [26,27,28]. Lycopene has shown potent antioxidant capacity and protective effect on cells by the suppression of oxidative damage [25]. It can also reduce the levels of inflammatory biomarkers in cells through its antioxidant activity [29]. Lycopene reportedly inhibits LPS-induced liver injury by reducing the serum levels of cytokines (IL-6 and tumor necrosis factor (TNF)-α) and increasing superoxide dismutase activity in mice [30]. Zou et al. [31] showed that lycopene suppresses IL-6 expression in LPS-stimulated macrophages by inhibiting the ROS-induced trafficking of TLR4 to lipid raft-like domains. Recently, we showed that lycopene inhibits NADPH oxidase activity and thus reduces ROS levels, leading to inhibition of NF-kB activation, IL-6 expression in pancreatic acinar cells stimulated with ethanol and palmitoleic acid [32]. Jhou et al. [33] demonstrated that lycopene downregulates NADPH oxidase 4 protein expression of human liver adenocarcinoma SKHep-1 cells. Lycopene decreased oxidative stress partly by downregulation of the expression of NADPH oxidase subunits in hepatic stellate cells [34]. Therefore, lycopene may have an inhibitory effect against HDM-induced inflammation by suppressing ROS production and TRL4 activation in respiratory epithelial cells. 

The current study aimed to investigate whether HDM induce the expression of IL-6 and IL-8 through TLR4 signaling and intracellular and mitochondrial ROS production, and whether lycopene inhibits HDM-induced IL-6 and IL-8 production by suppressing TLR4 activation and reducing ROS levels in A549 cells.

## 2. Results

### 2.1. HDM Induce IL-6 and IL-8 Expression and Increase ROS in A549 Cells

**Figure 1:**
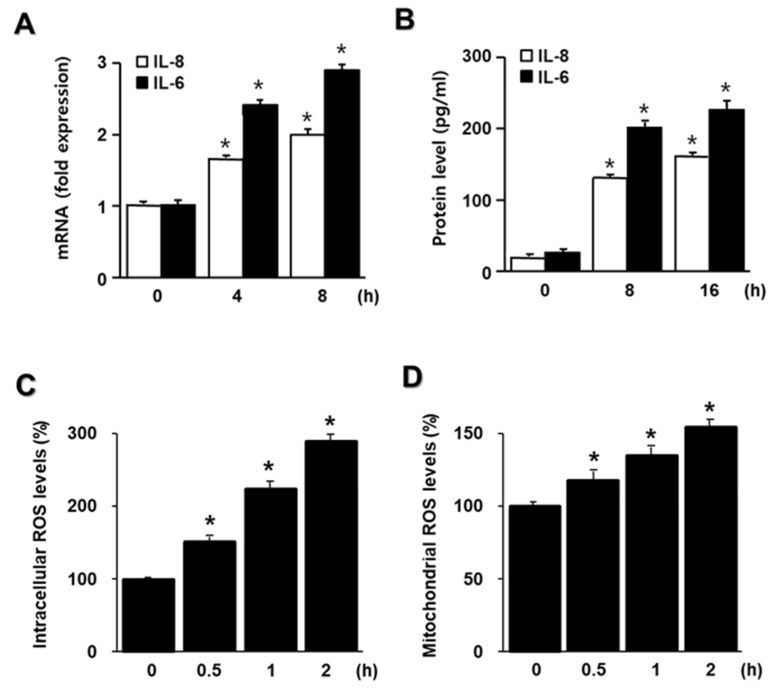
IL-6 and IL-8 mRNA and protein expression and intracellular and mitochondrial levels of ROS upon treatment with house dust mites (HDM). Cells were treated with 20 μg/mL HDM for the indicated time periods. (**A**) mRNA expression of IL-6 and IL-8 was determined with reverse transcription-polymerase chain reaction (RT-PCR) analysis. The mRNA levels at 0 h were set as 1. (**B**) IL-6 and IL-8 protein levels in the medium were determined with enzyme-linked immunosorbent assay (ELISA). (**C**) The intracellular ROS levels were determined using DCF-DA. ROS levels of the cells at 0 h were set as 100%. (**D**) The mitochondrial ROS levels were determined using MitoSOX. ROS levels of the cells at 0 h were set as 100%. All data are shown as the mean ± S.E. of three independent experiments. * *p* < 0.05 versus 0 h.

To investigate whether HDM induce IL-6 and IL-8 mRNA expression, cells were incubated with 20 μg/mL HDM for the indicated time periods (Figure 1A). As shown in Figure 1A, both IL-6 and IL-8 mRNA expression increased the most at 8 h. To determine whether the increase in IL-6 and IL-8 mRNA levels was followed by increased IL-6 and IL-8 protein production due to stimulation by HDM, their levels in the culture medium were determined following the treatment of cells with HDM for the indicated time periods. As shown in Figure 1B, the release of IL-6 and IL-8 showed the highest increase at 16 h of HDM treatment compared to that in cells at 0 h. The results indicated that HDM induced IL-6 and IL-8 production at both mRNA and protein levels in human respiratory epithelial A549 cells. 

For time-course experiment on intracellular and mitochondrial ROS levels, the cells were incubated with 20 μg/mL HDM for the indicated time periods (Figure 1C,D). Both intracellular and mitochondrial ROS levels increased time-dependently and are the most at 2 h. Thus, for the studies on the effect of lycopene on ROS levels, the cells were pretreated with lycopene and stimulated with HDM for 2 h. 

### 2.2. Lycopene Inhibits HDM-Induced Expression of IL-6 and IL-8 in A549 Cells

**Figure 2:**
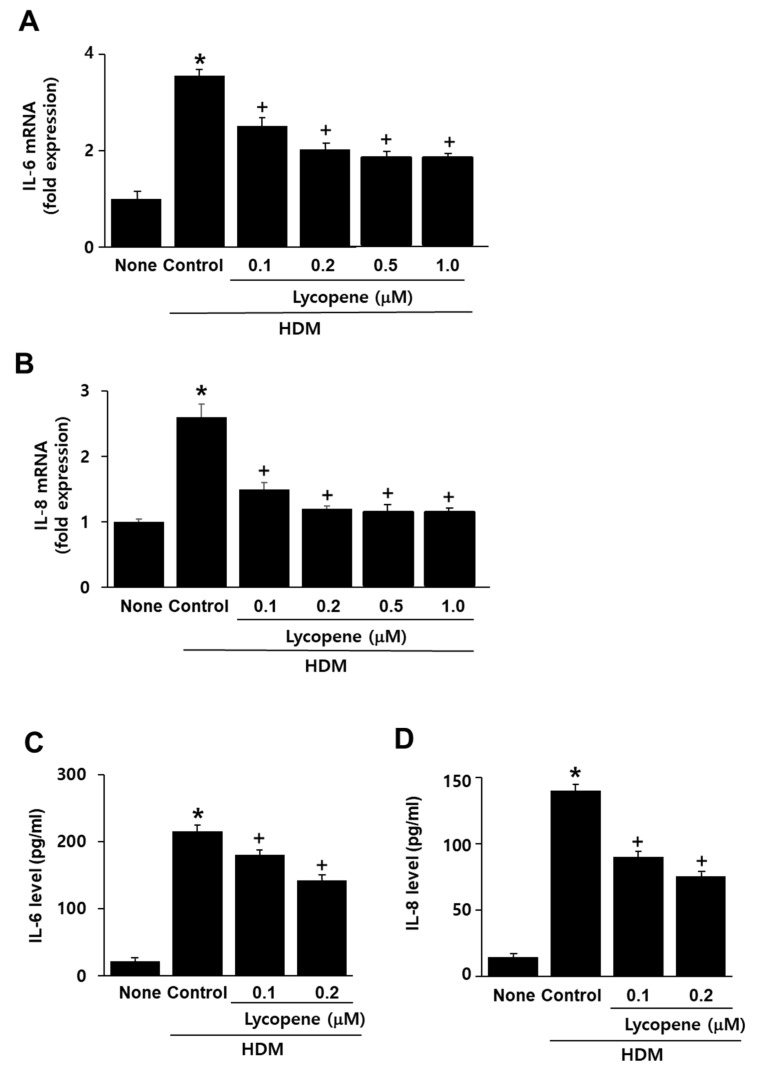
Effects of lycopene on the expression of IL-6 and IL-8 in house dust mite (HDM)-stimulated A549 cells. Cells were pretreated with lycopene (at the indicated concentrations) for 1 h, and then with HDM (20 μg/mL) for 8 h (for determination of mRNA expression) or 16 h (for determination of protein levels). (**A**,**B**) mRNA expression was determined with reverse transcription-polymerase chain reaction (RT-PCR) analysis. mRNA levels of the cells without any treatment (none) were set as 1. (**C**,**D**) The protein levels of IL-6 and IL-8 in the medium were determined using enzyme-linked immunosorbent assay (ELISA). All data are shown as the mean ± S.E. of three independent experiments. * *p* < 0.05 vs. none (cells without any treatment); + *p* < 0.05 vs. control (cells with HDM alone).

To determine its appropriate concentration, four concentrations of lycopene (0.1, 0.2, 0.5, and 1 μM) were used. As shown in Figure 2A,B, the HDM-induced mRNA expression of IL-6 and IL-8 was suppressed by lycopene. The inhibitory effect of 0.2 μM lycopene on cytokine mRNA expression was higher than that of 0.1 μM lycopene. The effect of 0.2 μM lycopene on cytokine mRNA expression was similar to that of 0.5 μM and 1.0 μM lycopene. Thus, for the studies on the effects of lycopene on cytokine protein levels, ROS levels, and TLR4 activity, HDM-stimulated cells were treated with 0.1 and 0.2 μM lycopene.

To investigate whether lycopene inhibits HDM-induced IL-6 and IL-8 protein expression, cells were pretreated with lycopene (0.1, 0.2 μM) and then with HDM. As shown in Figure 2C,D, the HDM-induced IL-6 and IL-8 protein expression was suppressed by lycopene treatment.

### 2.3. Lycopene Inhibits HDM-Induced Production of Intracellular and Mitochodrial ROS in A549 Cells

**Figure 3:**
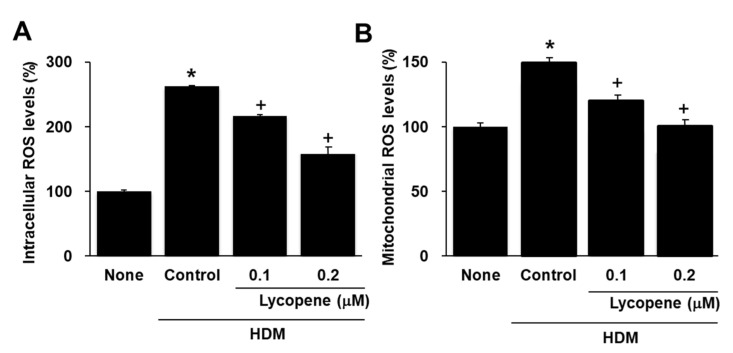
Effects of lycopene on the intracellular and mitochondrial levels of ROS in house dust mite (HDM)-stimulated A549 cells. Cells were pretreated with lycopene (0.1, 0.2 μM) for 1 h and then with HDM (20 μg/mL) for 2 h. (**A**) The intracellular ROS levels were determined using DCF-DA. ROS levels of the cells without any treatment (none) were set as 100%. (**B**) The mitochondrial ROS levels were determined using MitoSOX. ROS levels of the cells without any treatment (none) were set as 100% All data are shown as the mean ± S.E. of three independent experiments. * *p* < 0.05 vs. none (cells without any treatment); + *p* < 0.05 vs. control (cells with HDM alone).

To determine whether lycopene inhibits the HDM-induced production of intracellular ROS and mitochondrial ROS, cells were pretreated with lycopene and then with HDM. The results obtained from the dichlorofluorescein diacetate- and MitoSOX-based assays are shown in Figure 3A,B. The intracellular ROS levels in the cells treated with HDM (“Control”) were increased by 150% compared to those of untreated cells (“None”), whereas the levels of ROS in the mitochondria were increased by 40%. Pre-incubation of the cells with lycopene reduced the HDM-induced increase in ROS, which at 0.2 µM lycopene corresponded to a 40% reduction in intracellular ROS (Figure 3A) and a 30% reduction in mitochondrial ROS levels (Figure 3B).

### 2.4. Lycopene Inhibits HDM-Induced Toll Like Receptor 4 (TLR4) Activation in A549 Cells

**Figure 4:**
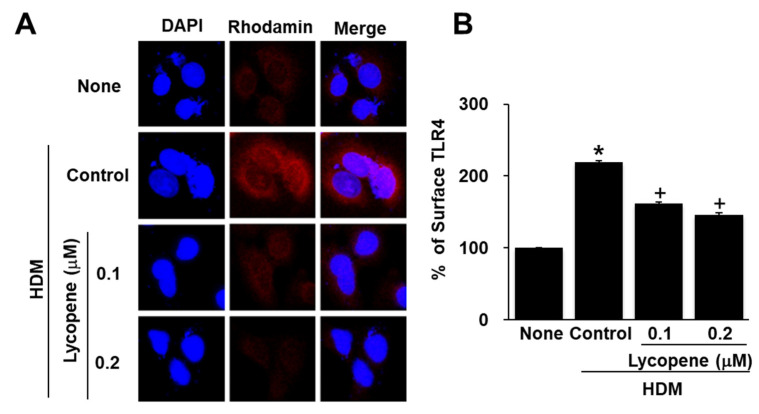
Effect of lycopene on TLR4 Activation in house dust mite (HDM)-stimulated A549 Cells. Cells were pretreated with lycopene (0.1 and 0.2 μM) for 1 h and then with HDMs (20 μg/mL) for 2 h. (**A**) The cells in slides were immunostained with a polyclonal antibody for TLR4. The immunoreactive proteins were visualized using a rhodamine-conjugated mouse anti-rabbit IgG antibody with DAPI counter-staining of the same field. (**B**) Surface TLR4 levels were examined by measuring the monoclonal anti-TLR4-MD-2 antibody conjugated with phycoerythrin (PE), using flow cytometry, and the mean fluorescence intensity was calculated thereafter. Surface TLR4 levels of the cells without any treatment (none) were set as 100%. All data are shown as the mean ± S.E. of three independent experiments. * *p* < 0.05 vs. none (cells without any treatment); + *p* < 0.05 vs. control (cells with HDM alone).

Next, we investigated whether HDM-induced TLR4 activation is inhibited following pretreatment with lycopene. A459 cells were treated with HDM and the level of TLR4 was examined by immunofluorescence using a polyclonal antibody for TLR4. and a rhodamine-conjugated mouse anti-rabbit IgG antibody with DAPI counter-staining. As shown in Figure 4A, HDM-treatment increased the level of TLR4 and this effect was significantly inhibited in cells pretreated with lycopene. The results show that HDM may interact with TLR4. TLR4 is associated with the adapto protein myeloid differentiation protein 2 (MD-2). Thus, we examined the effect of HDM and lycopene on the cell surface level of TLR4 by measuring the monoclonal anti-TLR4-MD-2 antibody conjugated with phycoerythrin (PE), using flow cytometry (Figure 4B). Fluorescence analysis show that HDM stimulation increased surface TLR4 levels; this increase was prevented by lycopene treatment in A549 cells. These results indicate that HDM stimulation induces TLR4 activation, which is eventually inhibited by lycopene. 

### 2.5. TAK242 Inhibits the HDM-Induced mRNA Expression of IL-6 and IL-8 and Intracellular and Mitochondrial ROS Increase in A549 Cells

**Figure 5:**
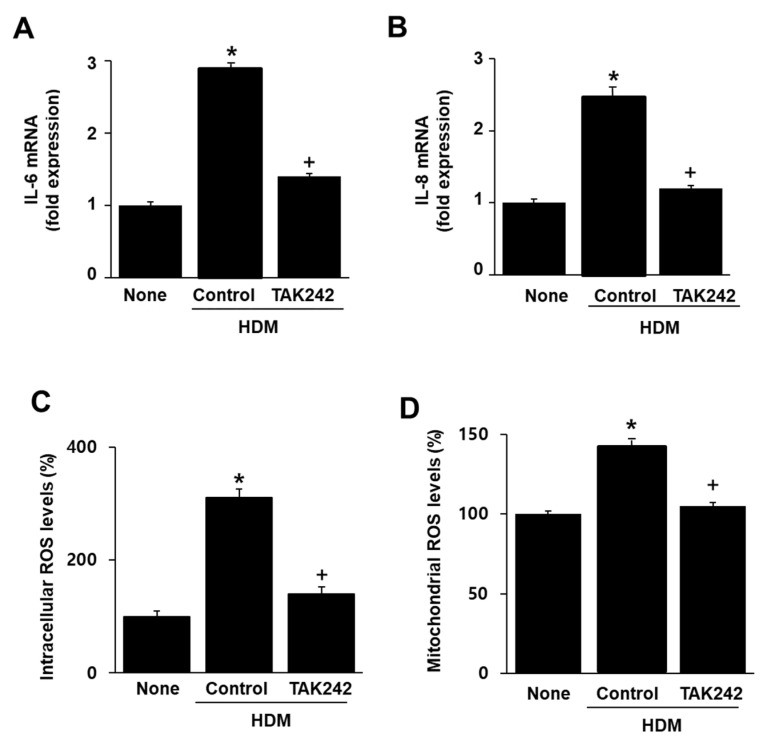
Effects of TAK242 on the mRNA expression of IL-6 and IL-8 and intracellular and mitochondrial ROS levels in human dust mite (HDM)-stimulated A549 cells. (**A**,**B**) Cells were pretreated with TAK242 (0.5 μM) for 1 h and then treated with HDM (20 μg/mL) for 8 h. mRNA expression of IL-6 and IL-8 was determined with RT-PCR analysis. The mRNA levels in cells without any treatment (none) were set as 1. (**C**,**D**) Cells were pretreated with TAK242 (0.5 μM) for 1 h and then treated with HDMs (20 μg/mL) for 2 h. (**C**) Intracellular ROS levels were determined using DCF-DA. ROS levels in the cells without any treatment (none) were set as 100%. (**D**) Mitocondrial ROS levels were determined using MitoSOX. ROS levels in the cells without any treatment (none) were set as 100%. All data are shown as the mean ± S.E. of three independent experiments. * *p* < 0.05 vs. none (cells without any treatment); + *p* < 0.05 vs. control (cells with HDM alone).

To investigate whether the effect of HDM on IL-6 and IL-8 mRNA expression are mediated through the TLR4 receptor, cells were pretreated with TAK242, a TLR4 inhibitor, and then treated with HDM. TAK242 suppressed HDM-induced IL-6 and IL-8 mRNA expression (Figure 5A,B). This indicates that TLR4 mediates HDM-induced IL-6 and IL-8 mRNA expression.

To investigate whether TLR4 mediates HDM-induced intracellular and mitochondrial ROS production, the cells were pretreated with TAK242 and then treated with HDM. As shown in Figure 5C,D, HDM induced an increase in intracellular and mitochondrial ROS levels, which were subsequently suppressed by treatment with TAK242. These results indicated that TLR4 mediated HDM-induced increase in intracellular and mitochondrial ROS.

## 3. Discussion

Exposure to HDM-allergens induced ROS production in the respiratory epithelial cells of patients with asthma [35]. In airway inflammation, ROS, generated by several inflammatory cells and oxygen metabolites, contributed to epithelial damage. ROS are considered the primary cause of bronchoconstriction, mucus secretion, and increased airway responsiveness [36]. 

Wang et al. [37] showed that the HDM allergen Der f 1 (Group 1 allergen of *D. farinae*) induces the release of cytokines (IL-25 and IL-33) in airway epithelial cells. Der f 1 increases ROS production and activates mitogen-activated protein kinases (MAPKs) to induce IL-8 expression in human basophilic cells [38]. Der f1 increased ROS production in neutrophils isolated from asthmatic versus non-asthmatic subjects [35]. In a previous study, the intratracheal administration of the HDM allergen Der p 2 (Group 2 allergen of D. *pteronyssinus*) to mice led to inflammatory cell infiltration, mucus gland hyperplasia in the bronchial epithelium, and elevated ROS in bronchoalveolar lavage fluids [39]. Der p2 induced nerve growth factor release and increases ROS levels in LA4 lung epithelial cells [40]. Although the HDM allergens were different, these studies demonstrate the critical role of ROS in HDM-induced respiratory inflammation.

In the present study, we used a standardized extract of D. *farinae* from the Arthropods of Medical Importance Resource Bank (AMIB), Yonsei University (Seoul, Korea). Major allergens are present at a concentration of 17.0 µg/mg (5.0 µg/mg of Der f 1 and 12.0 µg/mg of Der f 2), with the allergy unit (AU) of 12.5 AU/µg for the *D. farinae* extract [41]. Further studies should be performed to determine the effect of lycopene on TLR4 signaling and inflammatory cytokine expression in respiratory epithelial cells exposed to different allergens from HDM, such as Der f 1, Der f 2, Der p 1, and Der p 2, rather than the extract.

Regarding cytokine expression, Jang et al. [42] found that HDM (Der p1) induced the mRNA expression of IL-6 and IL-8 ten times more than TNF-α in THP-1 human monocytic cells. Shi et al. [43] examined the effect of HDM allergen Der p1 on proinflammatory cytokine production (TNF-α, IL-1β, IL-6, IL-8) in cultured primary nasal epithelial cells (NECs) from patients with allergic rhinitis (AR) and control NECs. They found significantly elevated IL-6 and IL-8 production in both NECs after Der p1 stimulation. The levels of IL-6 and IL-8 were 20–100 times higher than that of IL-1β in Der p1-stimulated cells at 24 h-culture. The level of TNF-α was six times higher than that of IL-1β after 24 h-stimulation of HDM. Wong et al. [44] showed that HDM induced the release of IL-6, IL-8, and TNF-α in eosinophils and bronchial epithelial BEAS-2B cells. After 18 h of stimulation with HDM, the levels of IL-6 and IL-8 were seven to eight times higher than that of TNF-α These studies suggest that IL-6 and IL-8 may have critical roles in HDM-associated inflammation compared to TNF-α and IL-1β. Therefore, we assayed for the levels of IL-6 and IL-8 in HDM-stimulated respiratory cells to determine the effects of lycopene. Future studies should determine the expression of various inflammatory cytokines, including TNF-α, MCP-1, and IL-1, β, in cells stimulated with different allergens of HDM. 

For the role of the redox-sensitive transcription factor in cytokine expression, Wong et al. [44] demonstrated that the Der p1-induced expression of cytokines is mediated by NF-κB in eosionphils and BEAS-2B cells. We recently showed that the HDM extracts induce the activation of mitogen-activated protein kinases (MAPKs), NF-κB κ, and activator protein-1 in human respiratory epithelial H292 cells [4]. Ascorbic acid suppresses the ROS-mediated activation of MAPKs and transcription factors (NF-κB κ and AP-1) by reducing ROS levels in H292 cells. Therefore, we postulate that lycopene may inhibit these inflammatory signaling pathways and NF-κB, since it decreases ROS levels in HDM-stimulated cells. The anti-inflammatory mechanisms of lycopene should be further examined in HDM-stimulated cells by examining the activities of NF-κΒ, AP-1, and MAPKs.

Regarding the role of intracellular and mitochondrial ROS in airway inflammation, direct exposure of the human bronchial epithelial cells BEAS-2B to HDM extracts resulted in increased cellular ROS production, mitochondrial oxidative stress, and nitrosative stress [45]. Lowe et al. [46] showed that the mitochondria-targeted antioxidant SS31 reduced airway inflammation in HDM-stimulated mice. They suggested that mitochondrial oxidative stress may contribute to airway hyperresponsiveness and inflammation. These studies support the present findings that HDM (extract of D. *farinae*) increased both intracellular and mitochondrial ROS levels as well as the expression of IL-6 and IL-8 in respiratory epithelial A549 cells. 

HDM induced TRL4 activation and subsequently increased the levels of innate pro-allergic cytokines, granulocyte-macrophage colony stimulating factor, IL-25, and IL-33 in airway structural cells [7]. Additionally, HDM increased TLR 4-mediated infiltration of eosinophils and neutrophils into the lungs of mice [47]. The HDM allergen Derp 2 activated TLR4 and induced IL-6, IL-8, and MCP-1 in THP-1 cells and lymphocytes [48]. These studies demonstrate the relationship between TLR4 and cytokine expression in HDM-stimulated cells. 

Upon stimulation, TLR4 is recruited to lipid rafts and subsequently interacts with its adaptor molecules, leading to the activation of downstream targets, such as MAPKs and NF-κB, and the production of pro-inflammatory cytokines in RAW264.7 macrophages [49,50]. This process occurs in an ROS-dependent manner because the inhibition of NADPH oxidase suppresses TLR4 recruitment to lipid rafts [51]. As previously mentioned, Hristova et al. [10] demonstrated that NADPH oxidase DUOX 1 has important role to produce ROS and activate TLR4 in HDM-stimulated airway epithelium. These studies suggest that NADPH oxidase-mediated ROS may be important to recruit TLR4 to lipid rafts and interact with its adaptor proteins for TLR4 signaling. 

Zou et al. [31] suggested that lycopene may prevent LPS-induced TLR4 assembly into lipid rafts by reducing intracellular ROS levels in macrophages. Thus, reducing ROS through lycopene may suppress TLR 4 recruitment into lipid rafts and inhibit the activation of downstream signaling pathways, such as MAPKs and NF-κB. Previously, we and others showed that lycopene inhibits NADPH oxidase activity in various cells [32,33,34]. Therefore, lycopene may inhibit NADPH oxidase in HDM-stimulated cells. Further studies are required to determine whether lycopene suppresses NADPH oxidase and TLR4 recruitment into lipid rafts in the HDM-stimulated cells.

Jiang et al. [34,52] demonstrated that TLR4 is upstream signaling for ROS production and expression of inflammatory cytokines (IL-1β, IL-6 and TNF-α) in lung tissues of LPS-stimulated mice and LPS-stimulated RAW 264.7 macrophages. 

Here, we found that lycopene inhibited the activation of TLR4 by reducing the surface levels of TLR4, which increased by HDM stimulation of the cells. TAK242, a TLR4 inhibitor, which disrupts the interaction of TLR4 with adaptor molecules [53], suppressed both intracellular and mitochondrial ROS production and the expression of IL-6 and IL-8 in HDM-stimulated A549 cells. The previous studies and the present finding suggest that ROS activate TLR4 and TLR4 activation increases ROS levels. Therefore, inhibitory effect of TLR4 activation may be caused by antioxidant activity of lycopene in the present study.

Lipid rafts are cell-membrane microdomains composed of cholesterol and sphingolipids, such as monosialotetrahexosylgangliside (GM1), which form a separate liquid-ordered phase in the liquid-disordered matrix of the cell membrane lipid layer [54,55]. Thus, it is essential to determine whether lycopene affects TLR4 localization within membrane lipid rafts by regulating the levels of GM1 in lipid rafts. In addition, it may be necessary to determine the effect of lycopene in the absence or presence of TAK242 to evaluate the synergistic effect of the co-treatment with lycopene and TAK242 in the present system.

Anathy et al. [56] demonstrated that HDM induced mitochondrial fission by increasing fission protein dynamin related protein 1 in human bronchial epithelial cells at 80 min-culture. They showed that HDM exposure increased endoplasmic reticulum-mitochondrial interactions and subsequent mitochondrial fission in bronchial epithelial cells. Therefore, HDM may directly attack mitochondria and mitochondrial dysfunction, which increases mitochondrial ROS.

Lycopene is a potent antioxidant carotenoid that can efficiently quench singlet oxygen species [57]. Tomato and tomato-based products account for 80% of lycopene intake in western countries. Watermelon, pink grapefruit, apricot, and papaya also significantly contribute to lycopene intake [58]. Regarding its anti-inflammatory mechanism, lycopene can neutralize intracellular ROS, as well as reduce the secretion of pro-inflammatory cytokines by macrophages [59]. It decreases monocyte proliferation [60]. In addition, lycopene inhibits mitochondrial ROS production by protecting against stress-induced mitochondrial dysfunction nerve cells [61] and cardiomyocytes [62]. In particular, Zou et al. [31] demonstrated that lycopene suppresses the LPS-stimulated, TLR4-mediated induction of IL-6 expression in macrophages. 

Regarding ROS levels and cell viability, we previously showed that treatment with lycopene (0.25 μM) for 24 h reduces the viability (20% reduction in viable cell numbers) and intracellular ROS levels (50% reduction) of pancreatic cancer PANC-1 cells [63]. After 24 h of incubation, lycopene (0.5 μM) did not affect cell viability, but 1 μM lycopene induced apoptosis in gastric cancer AGS cells [64]; 0.5 μM lycopene slightly decreased ROS levels, while 1 μM lycopene significantly decreased ROS levels in AGS cells after 24 h of incubation [64]. 

Teodoro et al. [65] showed that lycopene (1, 3, and 5 μM) did not affect the viability of A549 cells after 48 h of incubation, but decreased the number of viable cells in three cancer cell lines, including breast, colon, and prostate cell lines (HT-29, T84, and MCF-7), after 48 h. These results showed that the effect of lycopene on cell viability is cell-type specific. Trejo-Solís et al. [66] demonstrated that lycopene treatment could selectively arrest cell growth and induce apoptosis in cancer cells without affecting normal cells. Based on these studies, we postulated that lycopene (0.1 and 0.2 μM) treatment for 16 h, which was used in the present study, may not affect cell viability in A549 cells. Further study should be performed to determine whether the treatment of A549 cells for different time periods with lycopene alone (0.1 and 0.2 μM) affects ROS levels and cell viability (2 h for ROS levels, 8 h for cytokine mRNA level, and 16 h for cytokine protein levels).

Regarding the time point for the determination of ROS levels and TLR4 activity, Zhang et al. [67] demonstrated that mixed HDM allergens increased ROS levels in Calu-3 human airway epithelial cells treated for 2.5 h, which was inhibited by TAK242. Ryu et al. [68] showed that HDM extracts increased the surface TLR4 levels and dual oxidase 2-mediated ROS production in nasal and bronchial epithelial cells after 1 h of incubation. The TLR4 activator CL 097 (2-(ethoxymethyl)-1H-imidazo(4,5-c)quinolin-4-amine) increased the intracellular ROS levels of airway epithelial cells following a 2.5 h incubation. These studies show that increases in ROS are parallel with TLR4 activation in HDM-stimulated respiratory epithelium. In the present study, both intracellular and mitochondrial ROS levels are the most at 2 h. Thus, TRL4 activity was determined after 2 h of stimulation with HDM. For further study, the time-course experiment on TLR4 activity in HDM-stimulated respiratory epithelial cells should be performed. 

**Figure 6:**
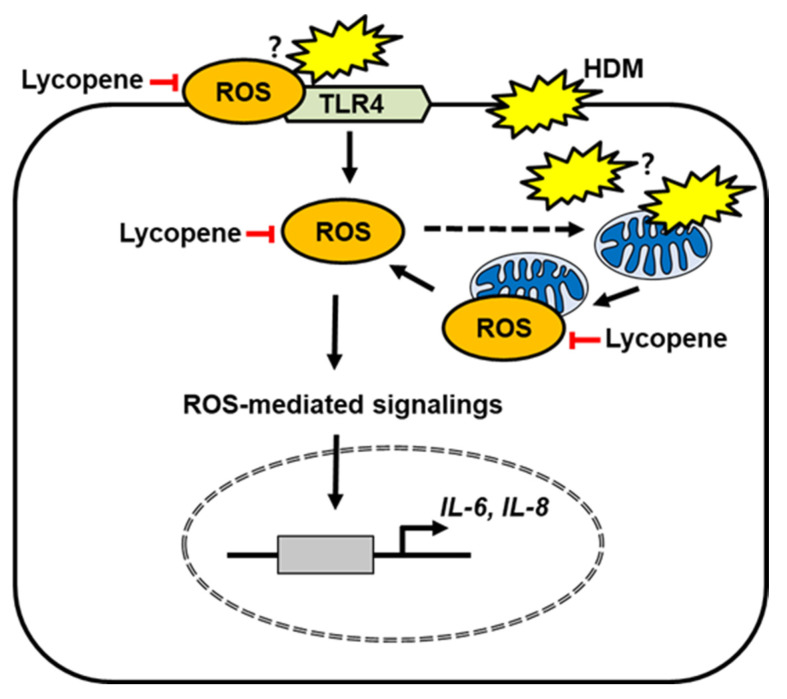
The proposed mechanism by which lycopene inhibits Toll like receptor 4 (TLR4)-mediated expression of inflammatory cytokines in house dust mites (HDM)-stimulated respiratory epithelial cells. HDM may increase the production of reactive oxygen species (ROS) and subsequent TLR4 activation, leading to increasing intracellular ROS levels in the cells. High amounts of ROS may cause mitochondrial dysfunction (dotted line) and increase mitochondrial ROS. In addition, HDM may directly attack mitochondria and increase mitochondrial ROS levels. ROS mediate inflammatory signalings, which induces expression of IL-6 and IL-8. Lycopene reduces ROS levels and inhibits TLR4 activation for cytokine expression. Lycopene scavenges mitochondrial ROS and subsequently suppresses expression of IL-6 and IL-8 in HDM-stimulated respiratory epithelial cells.

In the present study, lycopene reduced ROS levels and suppressed TLR4 activation in HDM-stimulated respiratory epithelial A549 cells. Since TLR4 activation leads to increases in intracellular ROS, lycopene may inhibit ROS-mediated inflammatory signaling to induce expression of IL-6 and IL-8 in HDM-stimulated cells. In addition, lycopene reduce mitochondrial ROS and prevents expression of IL-6 and IL-8 which induced by HDM stimulation (Figure 6). 

Based on the previous studies [6,56] showing that HDM enter the airway epithelium and induce mitochondrial fission, it is necessary to determine whether HDM induce mitochondrial dysfunction by directly interacting with mitochondria for further study. Since HDM stimulate NADPH oxidase in airway epithelial cells [10] and lycopene inhibits NADPH oxidase in various cells [32,33,34], it should be performed to determine whether lycopene inhibits NADPH oxidase in HDM-stimulated cells.

In conclusion, lycopene inhibits proinflammatory cytokine expression by suppressing HDM-induced increases in intracellular and mitochondrial ROS levels and TLR4 activation. Therefore, it may be considered beneficial for the prevention of respiratory airway inflammation caused by HDM. 

## 4. Materials and Methods

### 4.1. Reagents

The house dust mite *D. farinae* extract was purchased from the Arthropods of Medical Importance Resource Bank (AMIB), Yonsei University (Seoul, Korea). It was dissolved in PBS and stored at −80 °C. Lycopene (L9879, Sigma-Aldrich, St. Louis, MO, USA) was dissolved in tetrahydrofuran (THF). For the lycopene experiment, cells incubated with THF (less than 0.5%) alone served as the control. The TLR4 inhibitor, TAK242 (614316, Millipore, Burlington, MA, USA) was dissolved in dimethyl sulfoxide (DMSO). For the TAK242 experiment, cells incubated with DMSO (less than 0.3%) alone served as the control.

### 4.2. Cell Line and Culture Conditions

Human respiratory epithelial (A549) cells were cultured in RPMI-1640 medium (Gibco, Grand Island, NY, USA) supplemented with 10% heat-inactivated fetal bovine serum (Gibco, Grand Island, NY, USA), 100 units/mL penicillin, and 100 μg/mL streptomycin (Sigma-Aldrich). The cells were cultured at 37 °C in a humidified atmosphere of 5% CO_2_ and 95% air.

### 4.3. Quantitative Reverse Transcription-Polymerase Chain Reaction (qRT-PCR) Analysis for IL-6 and IL-8

The cells (6 × 10^4^ cells/well in 6 well plates) were cultured in the absence or presence of HDM for different durations or at different doses of HDM for 8 h. For the effect of lycopene, the cells were pretreated with lycopene (0.1, 0.2, 0.5, and 1.0 μM) or TAK242 (0.5 μM) for 1 h. The mRNA expression of IL-6 and IL-8 was assessed using qRT-PCR. Total RNA was isolated from the cells using TRI reagent (RNA/DNA/Protein isolation reagent, Molecular Research Center Inc., Cincinnati, OH, USA), and converted into cDNA by reverse transcription using a random hexamer and virus reverse transcriptase (Promega, Madison, WI, USA) at 23 °C for 10 min, 37 °C for 60 min, and 95 °C for 5 min. The cDNA was incubated with SYBR Green Real-time PCR Master Mix (Toyobo, Osaka, Japan) that contained 10 pg/mL of forward and reverse primers, and amplified using a Light Cycler PCR system (Roche Applied Sciences, Indinapolis, IN, USA). Resl-time PCR was conducted with the following specific primers for IL-6, IL-8, and GAPDH. The sequences of IL-6 primers were: 5-ACAAAT TCG GTACATCCTC-3 for the forward primer and 5-GCAGAATGAGATGAG TTGT-3 for the reverse primer. The sequences of IL-8 primers were: 5-ATGACTTCCAAGCTGGCCGTGGCT-3 for the forward primer and 5-TCTCAG CCCTCTTCAAAAACTTCT-3 for the reverse primer. For GAPDH, the forward primer was 5-GAAGGTGAAGGTCGGAGT-3 and the reverse primer was 5-GAAGATGGTGATGGGATT-3′. The cDNA was amplified by 40 cycles of denaturation at 95 °C for 30 s, annealing at 56 °C for 20 s, and extension at 72 °C for 30 s. 

### 4.4. Enzyme-Linked Immunosorbent Assay (ELISA)

The cells (6 × 10^4^ cells/well in 6 well plates) were pretreated with lycopene (0.1, 0.2 μM) or TAK242 (0.5 μM) and stimulated with HDM (20 μg/mL) for 16 h. The culture supernatants were centrifuged at 10,000× *g* for 5 min and then collected to measure IL-6 and IL-8 levels. The latter were determined using an enzyme-linked immunosorbent assay (ELISA) kit (R&D Systems, Inc., Minneapolis, MN, USA) following the manufacturer’s instructions.

### 4.5. Determination of Intracellular ROS and Mitochondrial ROS Levels 

For time-course experiment, the cells were treated with HDM (20 μg/mL) and culture for 2 h. For the effect of lycopene or TAK242, the cells were pretreated with lycopene or TAK242 for 1 h and treated with HDM (20 μg/mL) for 2 h. For the determination of intracellular ROS levels, the cells were loaded with 10 μg/mL dichlorofluorescein diacetate (DCF-DA; Sigma-Aldrich) for 30 min. The cells were then washed and scraped off with PBS. The fluorescence intensity of dichlorofluorescein (DCF) was measured using a Victor5 multi-label counter (PerkinElmer Life and Analytical Sciences, Boston, MA, USA) with excitation at 488 nm and emission at 525 nm. ROS trapped in the cells were expressed in terms of a relative increase.

For the determination of mitochondrial ROS levels, the cells were treated with 10 μM MitoSOX (Life Technologies, Grand Island, NY, USA) for 30 min, before being washed and scraped into phosphate-buffered saline (PBS). The intensity of MitoSOX fluorescence at 585 nm (excitation at 524 nm) was measured with a Victor 5 multi-label counter (PerkinElmer Life and Analytical Sciences, Boston, MA, USA).

### 4.6. Measurement of Surface TLR4 Level 

Cells (4 × 10^5^ cells/10-cm dish) were pretreated with lycopene (0.1 and 0.2 μM) for 1 h and then with HDM (20 μg/mL) for 2 h. The cells were washed, scraped off into 1 mL of PBS, and centrifuged at 3000× *g* for 5 min. After discarding the supernatants, the cells were resuspended in an ice-cold fluorescence-activated cell sorting (FACS) staining solution (2% FBS; 30 mL of PBS, 0.6 mL of FBS), treated with monoclonal anti-TLR4-MD-2 antibody conjugated with PE (12-9924, Bioscience, San Diego, CA, USA) at 4°C for 30 min, and then centrifuged at 3000 × *g* for 10 min. This antibody recognizes the complex, but neither TLR4 nor MD-2 alone. After discarding the supernatants, the cells were washed with 500 μL of ice-cold FACS staining solution for 10 min and resuspended in 1 mL of PBS. The fluorescence of PE was measured (488-nm excitation/561-nm emission) using a flow cytometer (FACSVerse, Becton Dickinson and Company, Franklin Lakes, NJ, USA). TLR4 staining represents the surface TLR4 levels.

### 4.7. Immunofluorescence Staining

To investigate the effect of lycopene on the HDM-induced TLR4 activation, cells were pretreated with lycopene for 1 h, before treatment with HDM for 2 h on glass slides, and then fixed with cold 100% methanol. The fixed cells were blocked for 1 h in a blocking solution and then incubated at 20–22 °C for 1 h with a primary antibody against TLR4 (1:100; SC-10741, Santa Cruz Biotechnology, Dallas, TX, USA). After washing with PBS, the cells were allowed to react with a rhodamine-conjugated mouse anti-rabbit IgG antibody (1:200; SC-2492, Santa Cruz Biotechnology) for 1 h. After removal of the excess of secondary antibody, the cells were washed with PBS and covered with Vectashield antifade mounting medium containing 4,6-diamidino-2-phenylindole (DAPI). The preparations were incubated for 30 min to allow for saturation with DAPI. The cells were stained with rhodamine-conjugated antibody and subsequently examined under a laser scanning confocal microscope (Zeiss LSM700, Carl Zeiss Inc., Thornwood, NY, USA) and photographed.

### 4.8. Statistical Analysis

One-way analysis of variance followed by the Newman–Keuls post hoc test was used for statistical analysis. All data are reported as the mean ± standard error of three independent experiments. For each experiment, the number of patients in each group was four (*n* = 4 per group). Statistical significance was set at *p* < 0.05.

## Data Availability

The data used to support the findings of this study are included in this article.

## Abbreviations

AU	Allergy unit
HDM	House dust mites
DAPI	4′,6-Diamidino-2-phenylindole
DCF	Dichlorofluorescein
DCF-DA	Dichlorofluorescein diacetate
DMSO	Dimethyl sulfoxide
ELISA	Enzyme-linked immunosorbent assay
FACS	Fluorescence-activated single cell sorting
LPS	Lipopolysaccharide
MCP-1	Monocyte chemoattractant protein-1
MD-2	Myeloid differentiation protein 2
NECs	Nasal epithelial cells
PAR-2	Protease activated receptor-2
PE	Phycoerythrin
qRT-PCR	Quantitative reverse transcription-polymerase chain reaction
ROS	Reactive oxygen species
TLR4	Toll-like receptor 4
THF	Tetrahydrofuran
TNF-α	Tumor necrosis factor-α
